# Supplementary material for: Infectious agents is a risk factor for myxomatous mitral valve degeneration: A case control study
Source: BMC Infect Dis. 2017 Apr 21;17:297. doi: 10.1186/s12879-017-2387-8 (PMC5399830; doi:10.1186/s12879-017-2387-8)
Supplement: Additional file 1: — Quantification of antigens of Borrelia burgdorferi, Mycoplasma pneumoniae and MMP 9 containing only the cases with cardiovascular disease associated. (DOCX 15 kb) [file 12879_2017_2387_MOESM1_ESM.docx › INFD-D-16-00809R4.pdf]

BMC Infectious Diseases

INFECTIOUS AGENTS IS A RISK FACTOR FOR MYXOMATOUS MITRAL VALVE DEGENERATION: A CASE CONTROL STUDY

--Manuscript Draft--

**Manuscript Number:**	INFD-D-16-00809R4
**Full Title:**	INFECTIOUS AGENTS IS A RISK FACTOR FOR MYXOMATOUS MITRAL VALVE DEGENERATION: A CASE CONTROL STUDY
**Article Type:**	Research article
**Section/Category:**	Bacterial and fungal diseases
**Funding Information:**	Support Foundation of São Paulo Research (2014/04383-4) — Dr. Marcos Gradim Tiveron

Abstract:

Background: The etiology of myxomatous mitral valve degeneration (MVD) is not fully understood and may depend on time or environmental factors for which the interaction of infectious agents has not been documented. The purpose of the study is to analyze the effect of Mycoplasma pneumoniae (Mp), Chlamydophila pneumoniae (Cp) and Borrelia burgdorferi (Bb) on myxomatous mitral valve degeneration pathogenesis and establish whether increased in inflammation and collagen degradation in myxomatous mitral valve degeneration etiopathogenesis.
Methods: An immunohistochemical test was performed to detect the inflammatory cells (CD20, CD45, CD68) and Mp, Bb and MMP9 antigens in two groups. The in situ hybridization was performed to detect Chlamydophila pneumoniae and the bacteria study was performed using transmission electron microscopy. Group 1 (n=20), surgical specimen composed by myxomatous mitral valve degeneration, and group 2 (n=20), autopsy specimen composed by normal mitral valve. The data were analyzed using SigmaStat version 20 (SPSS Inc., Chicago, IL, USA). The groups were compared using Student's t test, Mann-Whitney test. A correlation analysis was performed using Spearman's correlation test.. P values lower than 0.05 were considered statistically significant.
Results: By immunohistochemistry, there was a higher inflammatory cells/mm2 for CD20 and CD45 in group 1, and CD68 in group 2. Higher number of Mp and Cp antigens was observed in group 1 and more Bb antigens was detected in group 2. The group 1 exhibited a positive correlation between the Bb and MVD percentage, between CD45 and Mp, and between MMP9 with Mp. These correlations were not observed in the group 2. Electron microscopy revealed the presence of structures compatible with microorganisms that feature Borrelia and Mycoplasma characteristics.
Conclusions: The presence of infectious agents, inflammatory cells and collagenases in mitral valves appear to contribute to the pathogenesis of MVD. Mycoplasma pneumoniae was strongly related with myxomatous mitral valve degeneration. Despite of low percentage of Borrelia burgdorferi in MD group, this agent was correlated with myxomatous degeneration and this may occour due synergistic actions between these infectious agents likely contribute to collagen degradation.

**Corresponding Author:**	Marcos Gradim Tiveron, Ph.D., MD<br>Universidade de Sao Paulo<br>BRAZIL
**Corresponding Author Secondary Information:**	
**Corresponding Author's Institution:**	Universidade de Sao Paulo
**Corresponding Author's Secondary Institution:**	
**First Author:**	Marcos Gradim Tiveron, Ph.D., MD
**First Author Secondary Information:**	
**Order of Authors:**	Marcos Gradim Tiveron, Ph.D., MD

Powered by Editorial Manager® and ProduXion Manager® from Aries Systems Corporation

	Pablo Maria Alberto Pomerantzeff, Ph.D., MD. Full Professor
	Maria de Lourdes Higuchi, Ph.D., MD. Full Professor
	Marcia Martins Reis, Ph.D.
	Jaqueline de Jesus Pereira, Ms. student
	Joyce Kawakami
	Renata Ikegami
	Carlos Manuel de Almeida Brandão, Ph.D., MD
	Fabio Biscegli Jatene, Ph.D., MD. Full Professor
**Order of Authors Secondary Information:**	
**Response to Reviewers:**	Dear Editor,<br><br>I'm very flattered for own assessment. I performed the essential revisions and consider this paper potentially acceptable for publication in BMC Infectious Diseases.<br>The previous revisions are described bellow.<br><br><br>FIRST REVISION<br>Reviewer #1: The manuscript "INFECTIOUS AGENTS IS A RISK FACTOR FOR MYXOMATOUS MITRAL VALVE DEGENERATION: A CASE CONTROL STUDY" by Tiveron and others aims at demonstrating the presence and involvement of Mycoplasma pneumonia, Chlamydophila pneumoniae and Borrelia burgdorferi sensu lato bacteria in the etiopathogenesis of myxomatous mitral valve degeneration. The presence of the bacteria and inflammatory cells were studied using immunohistochemical staining, in situ hybridization, and transmission electron microscopy. Since the etiology of mitral valve degeneration is not completely understood, and it is well possible that microbial pathogens are involved in this process, this study is in principle warranted. Unfortunately, there are such shortcomings in the manuscript that prevent it from being accepted.<br><br>First and foremost, I am concerned with the alleged demonstration of Borrelia burgdorferi in the valve samples for the following reasons:<br><br>A-There is only one good description in the literature of a patient with Lyme endocarditis with mitral valve involvement (Hidri et al, Clin Microbiol Infect 2012) making the condition extremely rare<br>- Yes, I agree that is a paper that describes the Lyme endocarditis with mitral valve involvement. Our study is the first that demonstrated with more details the presence of antigens and products of bacteria in samples of myxomatous mitral valve degeneration.<br><br>B-Based on literature Lyme borreliosis is very rare in Brazil, yet the authors find Borrelia burgdorferi antigens in the mitral valves of both mitral valve degeneration patients and the controls.<br>- In Brazil, was describe the Baggio-Yoshinari Syndrome caused by spirochetes in its atypical and latent morphology, which gives rise to clinical manifestations similar to those observed in Lyme disease.<br><br>C-Immunohistochemical staining (or TEM) alone is not specific enough to reliably demonstrate the presence of Borrelia burgdorferi in the valves.<br>- We work with Immunohistochemical staining and TEM together to detect the antigens (IHC) and presence of bacteria and your products with TEM.<br><br>What the authors should now do is to use PCR to show that there indeed are Borrelia burgdorferi bacteria in the valve samples. This can be done using the paraffin embedded samples by deparaffinizing the samples. The authors should also perform Borrelia burgdorferi serology on the patient serum samples (if available). At the same time, also the presence of Mycoplasma pneumonia, Chlamydophila pneumonia in the valve samples can be analyzed using PCR-assays specific for these bacteria.<br>Without PCR based demonstration of the bacteria in the samples, the manuscript is not

Powered by Editorial Manager® and ProduXion Manager® from Aries Systems Corporation

fit for publication.

Other comments
-In places, the author refer to Mycoplasma pneumonia, Chlamydophila pneumonia and Borrelia burgdorferi as symbiotic bacteria, which they clearly are not
We refer this bacteria as symbiotic because of demonstration in previous studies that described this relations. In our study, we believe that symbiotic or synergistic actions between Mycoplasma pneumonia and Borrelia burgdorferi explain the correlation between myxomatous mitral valve degeneration and low presence of Borrelia burgdorferi in this valves.

-Was there an ethical approval for the study?
-The Bioethics Committee of the Clinical Hospital of Medical School of the University of Sao Paulo approved the study, approval no. 0029/04. A written informed consent was taken from all participants. This was described On Methods section, 2.2 Study design (Line 6, page 5).

-What is the specificity of the Borrelia burgdorferi antibody (ab34970)?
-The antibody ab34970 reacts with Borrelia burgdorferi (Lyme Disease) and have purity > 95% (http://www.abcam.com/abpromise).
-The abbreviation Bb for Borrelia burgdorferi is in places Bp.
- We agree with this orientation. It was corrected.

SECOND REVISION
This study demonstrated significantly greater presence of Mycoplasma pneumoniae in tissue sections of patients with myxomatous mitral degeneration; however, the distribution pattern of the antigen is quite strange. In addition, the conclusion in the abstract is far from the results of this study. This reviewer has some comments as follows:
Major comments.
1.There are high background staining or artifacts in panels of Figure 4C and 4E, which are very important positive findings of this study. The diffuse and massive staining pattern seems to be strange for the antigen expression.
- The results are exactly these. The brown in Fig. C and yellow in E demonstrate the Mycoplasma pneumoniae antigen presence by immunohistochemical reaction. These analysis were made by Aperio, a technology that eliminates the use of an optical microscope that mark the presence of antigen.
2.Results were positive for Mycoplasma pneumoniae, but negative for both Chlamydophilia pneumoniae and Borrelia burgdorferi. The authors should carefully and intensively revise results and conclusions in the abstract and the discussion section (page 13) based on the statistical analysis.
- The conclusion was revised according to results.
The presence of infectious agents, inflammatory cells and collagenases in mitral valves appear to contribute to the pathogenesis of MVD. Mycoplasma pneumoniae was strongly related with myxomatous mitral valve degeneration. Despite of low percentage of Borrelia burgdorferi in MD group, this agent was correlated with myxomatous degeneration and this may occour due synergistic actions between this infectious agents likely contribute to collagen degradation.
3.Quantitative real-time PCR should be performed to identify the presence of pathogens in tissue samples of the mitral valves.
- We are providing the quantitative real-time PCR in our analyses. Please, I would like to request more time to perform the real-time PCR.
4.Data in table 3 and figures 3A and 4A are mostly duplicated. The authors should obliterate redundant data presentation.
- We agree with this orientation. We will exclude the table 3

Minor comments.
1.Panels in figure 3 should have similar magnification, unless there was specific reason.
- The magnification is different due different size of some samples.
2.In figure 1, “mixoyd” should be “myxoid”.
- We agree with this orientation. It was corrected.

THIRD REVISION

Powered by Editorial Manager® and ProduXion Manager® from Aries Systems Corporation

	We are studing the reactions of borrelia and mycoplasma by the PCR and they are being positive.<br>However, to proceed to do the PCR-real time, we need to redo the primers, since they have to be much smaller. But the biggest problem that we are found is the need for patterns of staggered amounts of DNA specific to the primers we are going to use, which will serve as a guiding scale for the PCR-real time.<br>For Borrelia burgdorferi there are no kits with this specifications to buy. Thus, we are looking for to clone the borrelia and achieve a pattern of different titulations. For Mycoplasma pneumoniae, we are buying a ready kit, but takes time to arrive in Brazil.<br>This process is long and is not simple. Because of this, the current papers have not use much real time PCR for infectious agents, like intracellular agents.<br>So we think that our paper deserves to be published because it showed, with well established techniques (Histology, Immunohistochemistry, In situ hybridization and Transmission electron microscopy) the presence and influence of these bacteria specially the Mycoplasma pneumoniae, on the myxomatous mitral valve degeneration.<br>The results of this study will form a strong foundation for future research into the hypothesis that symbiotic actions of the bacteria play a key role in the development of myxoid degeneration of the mitral valve. More studies will be needed to confirm this causal relationship.

Powered by Editorial Manager® and ProduXion Manager® from Aries Systems Corporation

Manuscript
Click here to download Manuscript Manuscript - Final.docx
Click here to view linked References

INFECTIOUS AGENTS IS A RISK FACTOR FOR MYXOMATOUS MITRAL VALVE DEGENERATION: A CASE CONTROL STUDY

Marcos Gradim Tiveron, Ph.D., MD.[1], Pablo Maria Alberto Pomerantzeff, Ph.D., MD.[2], Maria de Lourdes Higuchi, Ph.D., MD.[3], Marcia Martins Reis, Ph.D[4], Jaqueline de Jesus Pereira[5], Joyce Kawakami[6], Renata Ikegami[7], Carlos Manuel de Almeida Brandao, Ph.D., MD.[8], Fabio Biscegli Jatene, Ph.D., MD[9].

Program in Thoracic and Cardiovascular Surgery, Faculty of Medicine, University of Sao Paulo, Brazil

[1]Fellow of Department of Heart Valve Surgery. Program in Thoracic and Cardiovascular Surgery, Faculty of Medicine, University of Sao Paulo, Brazil; mgtiveron@yahoo.com.br.

[2]Associate Professor in the Program in Thoracic and Cardiovascular Surgery, Medical School, University of Sao Paulo, Brazil; dcipablo@incor.usp.br.

[3]Director of Cardiac Pathology Laboratory in the Heart Institute of the Clinical Hospital, Medical School, University of Sao Paulo, Sao Paulo, Brazil; anplourdes@incor.usp.br.

[4]Biologist in Cardiac Pathology Laboratory in the Heart Institute of the Clinical Hospital, Medical School, University of Sao Paulo, Sao Paulo, Brazil; anpmarcia@incor.usp.br.

[5]Biomedical technician in Cardiac Pathology Laboratory in the Heart Institute of the Clinical Hospital, Medical School, University of Sao Paulo, Sao Paulo, Brazil; jaque09.biomed@gmail.com.

[6]Biomedical technician in Cardiac Pathology Laboratory in the Heart Institute of the Clinical Hospital, Medical School, University of Sao Paulo, Sao Paulo, Brazil; joycekawakami@hotmail.com.

[7]Biologist in Cardiac Pathology Laboratory in the Heart Institute of the Clinical Hospital, Medical School, University of Sao Paulo, Sao Paulo, Brazil; renata.ikegami@incor.usp.br.

[8]Cardiac Surgeon in the Heart Institute of the Clinical Hospital, Medical School, University of Sao Paulo, Sao Paulo, Brazil; carlos.brandao@incor.usp.br.

[9]Full Professor in the Program in Thoracic and Cardiovascular Surgery, Medical School, University of Sao Paulo, Brazil; fabio.jatene@incor.usp.br.

Please address correspondence to: Marcos G. Tiveron

e-mail adress: mgtiveron@yahoo.com.br (MG Tiveron)

Heart Institute of Clinical Hospital, Medical School, University of Sao Paulo, Av. Dr. Eneas de Carvalho Aguiar, 44, Zip code: 5403-000 - Sao Paulo (SP), Brazil

Abstract

Background: The etiology of myxomatous mitral valve degeneration (MVD) is not fully understood and may depend on time or environmental factors for which the interaction of infectious agents has not been documented. The purpose of the study is to analyze the effect of *Mycoplasma pneumoniae* (Mp), *Chlamydophila pneumoniae* (Cp) and *Borrelia burgdorferi* (Bb) on myxomatous mitral valve degeneration pathogenesis and establish whether increased in inflammation and collagen degradation in myxomatous mitral valve degeneration etiopathogenesis.

Methods: An immunohistochemical test was performed to detect the inflammatory cells (CD20, CD45, CD68) and Mp, Bb and MMP9 antigens in two groups. The in situ hybridization was performed to detect *Chlamydophila pneumoniae* and the bacteria study was performed using transmission electron microscopy. Group 1 (n=20), surgical specimen composed by myxomatous mitral valve degeneration, and group 2 (n=20), autopsy specimen composed by normal mitral valve. The data were analyzed using SigmaStat version 20 (SPSS Inc., Chicago, IL, USA). The groups were compared using Student's t test, Mann-Whitney test. A correlation analysis was performed using Spearman's correlation test.. P values lower than 0.05 were considered statistically significant.

Results: By immunohistochemistry, there was a higher inflammatory cells/mm2 for CD20 and CD45 in group 1, and CD68 in group 2. Higher number of Mp and Cp antigens was observed in group 1 and more Bb antigens was detected in group 2. The group 1 exhibited a positive correlation between the Bb and MVD percentage, between CD45 and Mp, and between MMP9 with Mp. These correlations were not observed in the group 2. Electron microscopy revealed the presence of structures compatible with microorganisms that feature Borrelia and Mycoplasma characteristics.

Conclusions: The presence of infectious agents, inflammatory cells and collagenases in mitral valves appear to contribute to the pathogenesis of MVD. *Mycoplasma pneumoniae* was strongly related with myxomatous mitral valve degeneration. Despite of low percentage of *Borrelia burgdorferi* in MD group, this agent was correlated with myxomatous degeneration and this may occour due synergistic actions between these infectious agents likely contribute to collagen degradation.

Keywords: mitral valve, myxomatous degeneration, Borrelia burgdorferi, Mycoplasma pneumoniae, Chlamydophila pneumoniae, metalloproteinases, inflammatory markers.

Abbreviations: Mp: *Mycoplasma pneumoniae,* Cp: *Chlamydophila pneumoniae,* Bp: *Borrelia burgdorferi*, MMP9: metalloproteinase 9, MVD: mitral valve degeneration, MVP: mitral valve prolapse, MD: myxomatous degeneration, CO: control, IHC: immunohistochemistry, ISH: *In situ* hybridization, TEM: transmission electron microscopy.

1. BACKGROUND

Myxomatous mitral valve degeneration (MVD) is the most common mitral valve disease in developed countries [1]. It occurs when the valve matrix is compromised due to an imbalance in the levels of acid mucopolysaccharides [2,3]. Mitral valve prolapse is initiated by MVD, which serves as an anatomical substrate affecting the valve matrix. The prevalence of this condition is estimated at 2-3% and affects 150 million people around the world [4].

The etiology of this valvopathy is not fully understood. Familial forms show autosomal dominant transmission with variable penetrance, which may depend on age or environmental factors [5,6]. In addition, inflammatory cells, autonomic imbalances of valve nerve endings and the action of metalloproteinases on collagen degradation can contribute to and enhance the degeneration of the valve matrix [7-9]. However, new contributing factors in the etiology of myxomatous degeneration should be sought to better understand this disease. Such knowledge could help to decrease postoperative recurrence, which occurs in approximately 69.2% of valve repair cases after 20 years of follow-up and is connected with degenerative progression [10]. Among potential contributing environmental factors, the participation of infectious agents in MVD pathogenesis has not been described in the literature as a causative factor; nevertheless, they may contribute to increasing the level of valve inflammation and collagen degradation. The presence of symbiotic bacteria is associated with tissue injury, as previously shown in studies analyzing atheromatous plaques in which proliferating *Mycoplasma pneumonia* (Mp) and *Chlamydophila pneumoniae* (Cp) bacteria led to inflammation, collagen degradation and vulnerable plaque formation [11,12]. *Borrelia burgdorferi*

(Bb) is a bacteria that causes Lyme disease and leads to cardiac manifestations in 4% to 10% of cases; it may co-infect with various bacteria, including *Mycoplasma pneumoniae* [13,14].

Based on this association between bacteria and tissue injury, the aim of this study was to analyze the presence and involvement of infectious agents in the increased inflammation and collagen degradation associated with the etiopathogenesis of myxomatous mitral valve degeneration.

2. METHODS

2.1 Valves studied

We analyzed 40 segments of mitral valve tissue, divided into 2 groups of 20 fragments each. Group 1 (myxomatous degeneration, MD) consisted of mitral valve fragments collected from patients undergoing replacement or mitral valve repair after mitral regurgitation with mitral valve prolapse (MVP). MVP is defined by the presence of a systolic murmur in the mitral focus by clinical examination, supplemented by a transthoracic echocardiogram showing bulging of one or both leaflets at least 2 mm apart on the mitral valve ring plane, regardless of its thickness. MVD was further confirmed by histopathology analysis. Group 2 (control, CO) included segments of the mitral valve posterior cusp without signs of MVD collected in a macroscopic examination of the valve during necropsy of cadaver patients.

The exclusion criteria for the MD group were other cardiovascular diseases with a surgical indication and valve reoperation; for the CO group, samples from patients with known congenital or acquired heart valve disease or with any macroscopic signs of MVP were excluded. Additional exclusion criteria for both groups were age of less than eighteen years old and the non-agreement of the patient or legal representative for participation in the study.

The 40 fragments were analyzed using the following techniques: immunohistochemistry to detect *Mycoplasma pneumoniae*, *Borrelia burgdorferi*, inflammatory mediators and MMP9 antigens;

in situ hybridization to detect *Chlamydophila pneumoniae*; and transmission electron microscopy to observe the infectious agents.

2.2 Study design (Figure 1)

This was an observational, analytical, case-control study. For each specimen, measuring approximately 2 x 2 cm^2, we prepared slices of 5-μm sections for histological analysis, immunohistochemistry and *in situ* hybridization (fixation in 10% formalin), and transmission electron microscopy (3% glutaraldehyde fixation). All the evaluations were performed at the Cardiac Pathology Laboratory in the Heart Institute of the Clinical Hospital of the Medical School of the University of Sao Paulo. The Bioethics Committee of the Clinical Hospital of Medical School of the University of Sao Paulo approved the study under the number 0029/04.

2.3 Histology and immunohistochemistry (IHC)

Five-micron serial sections of formalin-fixed, paraffin-embedded tissue were cut and Movat-stained for immunohistochemical detection of antigens from microorganisms (*Mycoplasma pneumonia* and *Borrelia burgdorferi*), inflammatory cells (CD20, CD45 and CD68) and metalloproteinase. The tissue sections were deparaffinized in xylene, and antigen retrieval was performed in 0.01 mol-g/L citrate buffer, pH 6.0, for 10 min in a microwave. The slides were allowed to stand for 20 min at room temperature. To block endogenous peroxidase, the tissue sections were saturated with 6% hydrogen peroxide for 8 minutes, followed by avidin-biotin blocking for 20 minutes, incubation with a protein block for 30 minutes (Dako, Carpinteria, CA, USA), and then with primary antibodies against *Mycoplasma pneumoniae* (1:100; rabbit clone 10MR54; Fitzgerald International Inc., Concord, MA, USA), *Borrelia burgdorferi* (1:300; rabbit clone ab34970, ABCAM), MMP-9 (1:3,200; rabbit clone RB1539P; Neo Markers Inc., Fremont, CA, USA), CD20 (1:1,000; clone L26 M0755; Dako, Carpinteria, CA, USA), and CD45 (1:125; clone VCHL M0742 Dako, CA, California, USA) overnight. The reaction was visualized using a 3,30-diaminobenzidine tetrahydrochloride solution. The positive controls were aneurysm sections for *Mycoplasma*

pneumoniae and MMP9, positive myocardium for *Borrelia burgdorferi* and amygdala for inflammatory cells.

2.4 *In situ* hybridization (ISH)

In situ hybridization was performed to detect Cp DNA (50 ng/µl) using the probe ACAACGGCTAGAAATCAATTATAAGACTGAAGTTGAGCATATTCGTGAGGGAGTGCAGA TTTAGATCATGGTGTCATTGCCCAAGGTTAAAGTCTACGT. For cell permeabilization, we used Tris / 10 mM EDTA pH 9.0, endogenous peroxidase blocking with 6% H_20_2 and reduction of non-specific proteins with protein blocker (CAS Block - Invitrogen, MA, USA). The double-stranded DNA was denatured in an oven at 95 ± 5 °C, and *in situ* hybridization was performed at 60 °C for 19 hours in an oven. The signal was amplified using the Genpoint kit (Dako, Carpinteria, CA, USA), and the reaction was visualized with 3,3-diaminobenzidine chromogen (Dako, Carpinteria, CA, USA). The probe was omitted for the negative control. Histological sections previously diagnosed as positive for *Chlamydophila pneumoniae* were used as positive controls for the reactions.

2.5 Transmission electron microscopy (TEM)

Mitral valve specimens were fixed in 3% glutaraldehyde and postfixed in 1% osmium tetroxide solution. They were then washed in saline and kept until the next day in 0.5% uranyl acetate at 4 °C. The fragments were dehydrated in an ascending series of ethanol and propylene oxide, followed by infiltration with a mixture of propylene oxide and araldite added to pure resin. The fragments were embedded in flexible molds containing silicone resin and polymerized for 48 h at 60 °C. Semi-thin samples (0.5 µm) were collected and stained with methylene blue with 1% borax and 1% azure II. After selecting the best blocks, 70 nm-thick sections were cut with an ultramicrotome and placed on 200 mesh copper screens. The screens were contrasted with uranyl acetate and 5% lead citrate for examination under a transmission electron microscope (JEOL JEM-1010; JEOL Ltd., Akishima, Tokyo, Japan).

2.6 Methods of analysis

After immunohistochemical and *in situ* hybridization processing, the slides were scanned using a Scanscope CS System apparatus (Aperio Technologies, Inc., CA, USA) with a 20x Olympus UPlanSApo lens and 20x/0.75 specification. This device was coupled to a scanner that generated image files in .svs format. The scanned images were analyzed using the Aperio ImageScope View Software program (Aperio Technologies, Inc., CA, USA). The Image Scope Software is a new technology that eliminates the use of an optical microscope. The samples were analyzed using this tool, and an algorithm for nuclear and membrane marking was selected. For inflammatory cell analysis (CD20, CD45, CD68), the total number of positive cells was divided by the area in mm^2, and the rate is expressed as the total number of positive cells/mm^2. When the presence of a bacterial antigen and matrix metalloproteinase (MMP9) was detected, we counted the quantity of antigen signals in μm^2 in an area 1 mm wide across the entire length of the valve.

2.7 Statistical analysis

The data were analyzed using SigmaStat version 20 (SPSS Inc., Chicago, IL, USA). The groups were compared using Student's t test for normally distributed variables; the results are expressed as the mean ± standard deviation. The Mann-Whitney test was used for unpaired variables with asymmetrical distributions. Parametric data were expressed as the mean and standard deviation of the sample, and the independent groups were compared by Student's t test and analysis of variance. Nonparametric data were represented by the median, lower quartile (25th percentile) and upper quartile (75th percentile). A correlation analysis of the asymmetrically distributed ordinal variables was performed using Spearman's correlation test. Spearman's correlation test was also performed in different groups to test whether the infectious agents exerted their effects in association with another agent, inflammatory cells, or MMP9. P values lower than 0.05 were considered statistically significant.

3. RESULTS

3.1 Studied population

Forty mitral valve samples with MVD or a normal appearance were included. In group 1, 14 (70%) patients were male, and 6 (30%) were female. The mean age was 67.4 ± 9.2 years. The intraoperative findings during surgery on the mitral valve were prolapse in 9 (45%) patients, ruptured chordae tendineae in 7 (35%) patients and prolapse associated with chordal rupture in 4 (20%) patients. According to the classification of Carpentier [15], the P2 segment was involved in 14 (70%) patients, the A2 segment in 1 (5%) patient, the A3 segment in 1 (5%) patient, the P1 and P2 segments in 1 (5%) patient, the P2 and P3 segments in 1 (5%) patient, the A2 and A3 segments in 1 (5%) patient, and the A2 and P2 segments in 1 (5%) patient (Table 1). In group 2, 11 (55%) patients were male, and 9 (45%) were female. The mean age was 67.6 ± 12.0 years. The cause of death was acute myocardial infarction in 5 (25%) cases, ischemic stroke in 4 (20%) cases, acute respiratory failure due to pneumonia in 3 (15%) cases, rupture of the ascending aorta during acute aortic dissection of the ascending aorta in 2 (10%) cases, pulmonary embolism in 1 (5%) case, hypertrophic cardiomyopathy in 1 (5%) case, ischemic cardiomyopathy in 1 (5%) case, acute pulmonary edema in 1 (5%) case, abdominal aortic aneurysm rupture in 1 (5%) case, and heart failure in 1 (5%) case (Table 2). The P2 segment was removed for analysis. None of the samples had signs of myxomatous degeneration in macroscopic analyses performed by two investigators, including the pathologist who performed the autopsies.

Table 1. Baseline characteristics of patients with myxomatous mitral valves.

Age, y	Gender	Clinical diagnosis / surgery	Cusp analyzed*
72	M	MD + chordal rupture	P2
51	F	MD + chordal rupture	A3
73	F	MD + chordal rupture	P2
72	F	MD + MVP + chordal rupture	P2
60	F	MD + MVP + chordal rupture	P2
59	M	MD + MVP	P1+P2
72	M	MD + MVP + chordal rupture	P2
73	M	MD + chordal rupture	A2
72	M	MD + chordal rupture	P2+P3
68	M	MD + chordal rupture	P2

68	M	MD + MVP	P2
73	M	MD + MVP	P2
79	M	MD + MVP	P2+A2
56	M	MD + MVP	P2
63	F	MD + MVP	A2+A3
69	F	MD + chordal rupture	P2
44	M	MD + MVP	P2
78	M	MD + MVP	P2
69	M	MD + MVP	P2
76	M	MD + MVP + chordal rupture	P2

MD: myxomatous degeneration, MVP: mitral valve prolapse.

* According to the Carpentier classification

Table 2. Baseline characteristics of the control patients.

Age, y	Gender	Cause of death	Post mortem interval, h
42	M	Acute myocardial infarction	6
59	M	Pulmonary thromboembolism	7
76	M	Ischemic stroke	3
66	M	Ischemic cardiomyopathy	6
82	M	Ischemic stroke	5
63	F	Dissection of the ascending aorta	9
79	F	Dissection of the ascending aorta	9
73	F	Acute respiratory failure - Pneumonia	10
75	M	Left Ventricular free wall rupture by acute myocardial infarction	5
54	F	Hypertrophic cardiomyopathy	9
74	F	Acute pulmonary edema	9
51	M	Acute myocardial infarction	10
52	M	Abdominal aortic aneurysm ruptured	12
80	M	Acute respiratory failure - Pneumonia	5
60	M	Acute myocardial infarction	6
61	F	Ischemic stroke	6
72	F	Ischemic stroke	8
84	F	Acute myocardial infarction	5
67	M	Acute respiratory failure - Pneumonia	14
82	F	Heart failure	9

3.2 Percentage of degenerated tissue

The tissues were subjected to Movat staining to determine whether myxomatous degeneration was present. More degenerated tissue was observed in group 1, with a mean of 54.6% ± 23.7% degenerated tissue, compared with group 2, with 35.5% ± 22.5% degenerated tissue (p = 0.013; 95% CI 4.3 to 33.9) (Figure 2).

3.3 Presence of inflammatory cells (Figure 3)

An analysis of $CD20^+$ (B lymphocyte marker) and $CD45^+$ (T lymphocyte marker) cells revealed significantly more positive cells/mm^2 in valves with myxomatous degeneration ($CD20^+$ cells: group 1 median = 17.8 (6.7 - 27.9), group 2 median = 4.6 (3.6 - 9.8), p = 0.007; $CD45^+$ cells: group 1 median = 17.3 (3.4 - 92.5), group 2 median = 2.8 (1.4 - 10.1), p = 0.008). An analysis of $CD68^+$ cells

(macrophage marker) revealed more positive cells/mm^2 in the control group (group 1 median = 38.7 (26.6 - 81.8), group 2 median = 70 (42.7 - 120.4), p = 0.098) (Figure 3A).

3.4 Identification of bacteria by IHC, ISH and TEM (Figure 4)

The numbers of *Mycoplasma pneumoniae*, *Chlamydophila pneumoniae* and *Borrelia burgdorferi* bacterial antigens are expressed as µm^2 antigens per 1 mm of analyzed valve segment length (µm^{2}Ag/1 mm). A significantly greater *Mycoplasma pneumoniae* antigen area was detected in the MD group. The median positive area in µm^{2}Ag/1 mm for *Mycoplasma pneumoniae* was 180,993 (24,856 - 387,477) in group 1 (valves with myxomatous degeneration) versus 7,970 (2,736-15,992) in group 2 (normal valves) ($p < 0.001$). Similarly, for *Chlamydophila pneumoniae*, group 1 presented a greater antigen area (median = 9,905 (4,716 - 16,912) versus 5,864 (2,382 - 8,692) in group 2; p = 0.2). By contrast, more *Borrelia burgdorferi* antigens were detected in the control group (group 1 median = 7,596 (3,203 - 13,519), group 2 median = 10,584 (7,223 - 15,974); p = 0.14) (Figure 4A). Electron microscopy revealed the presence of structures compatible with microorganisms with *Borrelia* and *Mycoplasma* characteristics. The microorganisms identified in the MD group were smaller and thinner than the bacteria found in the control group.

3.5 MMP9 analysis and correlation test (Figure 5)

The presence of MMP9 is expressed in µm^2 of antigens per 1 mm valve segment length. We observed a significantly greater antigen area in the myxomatous degeneration group (median = 389,844 (214,459 - 679,711) versus 144,397 (29,894 - 247,453) in group 2; $p < 0.001$) (Figure 5A).

The valves with myxomatous degeneration exhibited significant correlations between the quantity of bacterial antigens, inflammatory cells and MMP9: *Borrelia burgdorferi* was positively correlated with the MD percentage (r = 0.52 and p = 0.018), and *Mycoplasma pneumoniae* was positively correlated with CD45 (r = 0.51 and p = 0.02) and MMP9 (r = 0.45 and p = 0.04). These correlations were absent in the control group (Table 3).

Table 3. Correlations between bacterial antigens *versus* inflammatory cells *versus* MMP-9.

	MD		CO	
	r	p	r	p
Bb x % MD	0.52	0.018	0.15	0.5
CD45 x Mp	0.51	0.02	0.01	0.9
Mp x MMP9	0.45	0.04	0.17	0.6
Mp x % MD	-0.34	0.13	0.04	0.8
MMP9 x % MD	-0.02	0.91	0.4	0.07

MD: myxomatous degeneration, CO:control Bb: *Borrelia burgdorferi* Mp: *Mycoplasma pneumoniae* MMP9: metalloproteinase 9.

4. DISCUSSION

To the best of our knowledge, this is the first report describing the presence of bacteria in the pathogenesis of myxomatous degeneration. Our results also show increased expression of CD20, CD45 and MMP9 in MVD.

In our work, we identified enhanced *Mycoplasma pneumoniae* and *Chlamydophila pneumoniae* antigens in mitral valves with MD compared with controls, with a median of 180,993 (24,856 to 387,477) versus 7,970 (2,736 to 15,992) μm^2 Ag/1 mm valve segment extension ($p < 0.001$) and 9,905 (4,716 - 16,912) x 5,864 (2,382 - 8,692) μm^2 Ag/1 mm valve segment extension ($p = 0.2$), respectively. We also observed positive correlations between *Mycoplasma pneumoniae* and $CD45^+$ cells ($r = 0.51$ and $p = 0.02$) and MMP9 ($r = 0.45$ and $p = 0.04$). The presence of *Borrelia burgdorferi* antigens was positively correlated with the percentage of MD ($r = 0.52$ and $p = 0.018$). In previous studies, the presence of various symbiotic bacteria has been associated with the formation of tissue injury. In the aortic valve, *Chlamydophila pneumoniae* and *Mycoplasma pneumoniae* proliferation was associated with tissue calcification and aortic valve stenosis [16,17]. Roggerio et al. studied the relationship between *Mycoplasma pneumoniae* and *Chlamydophila pneumoniae* in aortic aneurysms and identified these bacteria in the adventitia of the aorta, concluding that infectious agents, acting symbiotically, can contribute to the development of aortic aneurysms, initiating inflammation and contributing to the disease process [18].

Both groups had an average age of approximately 67 years. The valve impairment in the control group can be explained by age-related progressive degeneration of the extracellular matrix through the action of collagenolytic enzymes secreted by interstitial cells that cause abnormalities in collagen [19].

Infectious agents are often present in cardiovascular disease, with roles for these pathogens in its genesis or prior to its inception reported in several papers. Recently, Mangini et al. detected the presence of *Mycoplasma pneumoniae, Chlamydophila pneumoniae* and *Borrelia burgdorferi* in hearts with dilated cardiomyopathy, including Chagas disease [20]. In the aortic valve, the proliferation of *Chlamydophila pneumoniae* and *Mycoplasma pneumoniae* was related to tissue calcification and aortic valve stenosis [21]. Inflammatory cells and metalloproteinases have also been associated with the MD process. Veinot et al. analyzed five valves with MD and observed an increase in markers of mast cells (CD117), suggesting the participation of an inflammatory process triggered by myxoid degeneration. $CD117^+$ cells are young cells identified in mitral valves that represent a degree of heart regeneration capacity against stress, trauma or ischemia [3]. Barth et al. analyzed the presence of $CD34^+$ fibrocytes in 15 myxoid valves and 10 valves without degeneration and found that these cells appear to be morphologically altered [7]. Rabkin et al. identified a role for MMP9, as well as other collagenases like MMP1, MMP2 and MMP13, in the breakdown of collagen in myxomatous degeneration [19]. The involvement of bacteria in the etiology of MVD has not previously been described. It is possible that the presence of such agents could lead to collagen degradation and increase local inflammation, facilitating the process of myxoid degeneration. This scenario warrants further investigation.

The presence of microorganisms as host symbionts is not well understood but is increasingly being studied. Scholars believe that microbial symbionts may fundamentally alter the host organism's physiology and sometimes impart new or optimized abilities that result in more adapted or healthier individuals [22]. When more than one microparasite species infects the same host, interactions between these parasites can exacerbate their symbiotic and immunological effects [23,24]. In this study, the valves in the control group exhibited more *Borrelia burgdorferi* antigens than the valves in

the MVD group. However, in the valves with MVD, the presence of *Borrelia burgdorferi* was associated with more myxomatous degeneration, and an interaction with *Mycoplasma pneumoniae* may have contributed to collagen degradation through a synergistic effect between these agents. Considering that we observed *Mycoplasma pneumoniae* and *Borrelia burgdorferi* in both groups but in different quantities and morphologies, these agents might also produce different responses with respect to collagen degradation. The presence of *Mycoplasma pneumoniae* can stimulate hyperactive macrophages, with subsequent cytotoxic T cell activation inducing fibroblast apoptosis [25]. In addition, the MVD group exhibited more *Mycoplasma pneumonia*, which positively correlated with gelatinase MMP9, an enzyme that participates in collagen degradation, as reported by other authors [9,19]. In our study, we also identified a greater number of $CD68^+$ cells in the valve segments of the control group. Rabkin et al. associated the presence of macrophages in normal mitral valves with a minor amount of proteolytic enzymes [19]. Though valves with MD had fewer $CD68^+$ cells, they still had higher quantities of B and T lymphocytes, including cytotoxic T lymphocytes that can release cytokines such as perforin and the granzina B leading to cell apoptosis and contributing to collagen degradation. It is possible that the $CD68^+$ cells function as protective factors against collagen degradation.

Scholars have suggested that *Borrelia burgdorferi* strains interact competitively with the host [26]. In a recent study, Devevey et al. analyzed three different strains of *Borrelia burgdorferi sensu strictu* in 36 rats and detected a strong and competitive inhibitory interaction among the strains. In all cases, the strain that infected first exhibited a fitness advantage, mainly because colonization of the rat tissue by subsequent strains was inhibited. This inhibition is most likely due to the production of specific antibodies against *Borrelia* protein antigens [27]. The presence of bacteria and their relationships with collagen degradation as well as increased local inflammation can facilitate myxomatous degeneration, and the synergistic actions of multiple symbionts seem to be a contributing factor in MVD pathogenesis.

We are aware of the limitations of our study, especially the restrictions of the methodological design, including the sample size. Another limitation of this work is that the control group included

cases with cardiovascular diseases such as myocardial infarction and aortic aneurysm, though these conditions did not appear to influence the results (Supplementary Material). Moreover, in the control patients, we identified several degeneration foci with histological features consistent with MD, though they were not enough to lead to a breakdown in the valve structure. The results of this study will form a strong foundation for future research into the hypothesis that symbiotic actions of the bacteria play a key role in the development of myxoid degeneration of the mitral valve. More studies will be needed to confirm this causal relationship.

5. CONCLUSION

The presence of infectious agents, inflammatory cells and collagenases in mitral valves appear to contribute to the pathogenesis of MVD. *Mycoplasma pneumoniae* was strongly related with myxomatous mitral valve degeneration. Despite of low percentage of *Borrelia burgdorferi* in MD group, this agent was correlated with myxomatous degeneration and this may occour due synergistic actions between this infectious agents likely contribute to collagen degradation.

DECLARATIONS

Abreviations:

Mp: *Mycoplasma pneumoniae*, Cp: *Chlamydophila pneumoniae*, Bb: *Borrelia burgdorferi*, MMP9: metalloproteinase 9, MVD: mitral valve degeneration, MVP: mitral valve prolapse, MD: myxomatous degeneration, CO: control, IHC: immunohistochemistry, ISH: *in situ* hybridization, TEM: transmission electron microscopy.

Ethics approval and consent to participate

The Bioethics Committee of the Clinical Hospital of Medical School of the University of Sao Paulo approved the study, approval no. 0029/04. A written informed consent was taken from all participants. Written consent for participating in the study and consent to publish was obtained from each participant prior to the tests.

Consent for publication

Not applicable”

Availability of data and materials

All relevant data are available in the tables and figures. The raw data supporting our findings can be found by contact with autor across e-mail (mgtiveron@yahoo.com.br).

Competing interests

The authors declare that they have no competing interests

Funding

The funding for this study was provided by FAPESP (Support Foundation of São Paulo Research) by protocol number 2014/04383-4.

Authors' contributions

MGT are responsible conducted the experiment, the data collection and data analysis assistance of MMR. PMAP and MLH are responsible for the design and conception of the experiment and interpretation of final results. Reviewed, revised, and edited the final manuscript. JK and RI conducted the immunohistochemistry and *in situ* hybridization experiment. JJP conducted the transmission electron microscopy experiment and analysis. MMR conducted the data analysis and interpretation. CMAB reviewed, revised, and edited the final manuscript. FBJ reviewed and revised the manuscript. All authors contributed to the final manuscript. All authors read and approved the final manuscript.

Acknowledgements

We thank Creusa Dal Bó for statistical review.

Authors’ information

All the authors are from Heart Institute (InCor), School of Medicine of University of Sao Paulo.

Marcos Gradim Tiveron is Cardiac Surgeon and Fellow of Department of Heart Valve Surgery.

Pablo Maria Alberto Pomerantzeff is cardiac surgeon specialized in valve diseases and is the director of the Surgical Unit of Valve Cardiopathy.

Maria de Lourdes Higuchi is a pathologist expert in electron microscopy, director of the Laboratory of Cardiac Pathology, where the project was developed.

Márcia Martins Reis is biologists specialized in cardiac diseases, expert in immunohistochemestry.

Jaqueline de Jesus Pereira is a Ms. student in Physiopathology.

Joyce Kawakami is Biomedical technician in Cardiac Pathology Laboratory.

Renata Ikegami is Biologist in Cardiac Pathology.

Carlos Manuel de Almeida Brandão is Cardiac Surgeon specialized in valve diseases.

Fabio Biscegli Jatene is a Cardiac Surgeon and Head Professor in the Program in Thoracic and Cardiovascular Surgery.

REFERENCES

[1] Criley JM, Lewis KB, Humphries JO, Ross RS. Prolapse of the mitral valve: clinical and cine-angiocardiographic findings. Br Heart J 1966; 28:488-496.

[2] Grande-Allen KJ, Griffin BP, Ratliff NB, Cosgrove DM, Vesely I. Glycosaminoglycan profiles of myxomatous mitral leaflets and chordae parallel the severity of mechanical alterations. J Am Coll Cardiol 2003; 42:271-277.

[3] Veinot JP, Prichett-Pejic W, Song J, et al. CD117-positive cells and mast cells in adult human cardiac valves - observations and implications for the creation of bioengineered grafts. Cardiovasc Pathol 2006; 15:36-40.

[4] Freed LA, Levy D, Levine RA, et al. Prevalence and clinical outcome of mitral-valve prolapse. N Engl J Med 1999; 341:1-7.

[5] Hayek E, Gring CN, Griffin BP. Mitral valve prolapse. Lancet 2005; 365:507-518.

[6] Movahed MR, Hepner AD. Mitral valvar prolapse is significantly associated with low body mass index in addition to mitral and tricuspid regurgitation. Cardiol Young 2007; 17:172-174.

[7] Barth PJ, Koster H, Moosdorf R. $CD34^+$ fibrocytes in normal mitral valves and myxomatous mitral valve degeneration. Pathol Res Pract 2005; 201:301-304.

[8] Hu X, Zhao Q. Autonomic dysregulation as a novel underlying cause of mitral valve prolapse: A hypothesis. Med Sci Monit 2011; 17:27-31.

[9] Mahimkar R, Nguyen A, Mann M, et al. Cardiac transgenic matrix metalloproteinase-2 expression induces myxomatous valve degeneration: a potential model of mitral valve prolapse disease. Cardiovasc Pathol 2009; 18:253-261.

[10] David TE, Armstrong S, McCrindle BW, Manlhiot C. Late outcomes of mitral valve repair for mitral regurgitation due to degenerative disease. Circulation 2013; 127:1485-1492.

[11] Higuchi ML, Reis MM, Sambiase NV, et al. Coinfection with *Mycoplasma pneumoniae* and *Chlamydia pneumoniae* in ruptured plaques associated with acute myocardial infarction. Arq Bras Cardiol 2003; 81:12-22.

[12] Higuchi ML, Santos MH, Roggério A, Kawakami JT, Bezerra HG, Canzian M. A role for archaeal organisms in development of atherosclerotic vulnerable plaques and myxoid matrices. Clinics 2006; 61:473-478.

[13] Krause PJ, Bockenstedt LK. Lyme disease and the heart. Circulation 2013; 127:e451-e454.

[14] Berghoff W. Chronic Lyme disease and co-infections: differential diagnosis. Open Neurol J 2012; 6:158-178.

[15] Muresian H. The clinical anatomy of the mitral valve. Clin Anat 2009; 22:85-98.

[16] Nystrom-Rosander C, Thelin S, Hjelm E, et al. High incidence of *Chlamydia pneumoniae* in sclerotic heart valves of patients undergoing aortic valve replacement. Scand J Infect Dis 1997; 29:361-365.

[17] Higuchi-Dos-Santos MH, Pierri H, Higuchi ML, et al. *Chlamydia pneumoniae* and *Mycoplasma* pneumoniae in calcified nodes of stenosed aortic valves. Arq Bras Cardiol 2005; 84:443-448.

[18] Roggério A, Sambiase NV, Palomino SAP, et al. Correlation of bacterial coinfection versus matrix metalloproteinase 9 and tissue inhibitor of metalloproteinase 1 expression in aortic aneurysm and atherosclerosis. Ann Vasc Surg 2013; 27:964-971.

[19] Rabkin E, Aikawa M, Stone JR, Fukumoto Y, Libby P, Schoen FJ. Activated interstitial myofibroblasts express catabolic enzymes and mediate matrix remodeling in myxomatous heart valves. Circulation 2001; 104:2525-2532.

[20] Mangini S, Higuchi ML, Kawakami JT, et al. Infectious agents and inflammation in donated hearts and dilated cardiomyopathies related to cardiovascular diseases, Chagas' heart disease, primary and secondary dilated cardiomyopathies. Int J Cardiol 2015; 178:55-62.

[21] Pierri H, Higuchi-Dos-Santos MH, Higuchi Ml, et al. Density of *Chlamydia pneumoniae* is increased in fibrotic and calcified areas of degenerative aortic stenosis. Int J Cardiol 2006; 108:43-47.

[22] Hussa EA, Goodrich-Blair H. It takes a village: Ecological and fitness impacts of multipartite mutualism. Annu Rev Microbiol 2013; 67:161-178.

[23] Mideo N. Parasite adaptations to within-host competition. Trends Parasitol 2009; 25:261–268.

[24] Alizon S, van Baalen M. Multiple infections, immune dynamics, and the evolution of virulence. Am Nat 2008; 172:E150-E168.

[25] Muhlradt PF. Immunomodulation by mycoplasmas: artifacts, facts and active molecules. In: Razin S, Herrmann R, editors. Molecular biology and pathogenicity of mycoplasma. New York: Kluwer Academic/Plenum Publishers, 2002:449.

[26] Pedersen AB, Fenton A. Emphasizing the ecology in parasite community ecology. Trends Ecol Evol 2007; 22:133-139.

[27] Devevey G, Dang T, Graves CJ, Murray S, Brisson D. First arrived takes all: inhibitory priority effects dominate competition between co-infecting Borrelia burgdorferi strains. BMC Microbiol 2015; 15:61.

FIGURE LEGENDS

Fig. 1. Study design.

Mp: *Mycoplasma pneumonia*; Bb: *Borrelia burgdorferi*.

Fig. 2. Histological analysis of myxoid degeneration. A: analysis of myxomatous degeneration percentage (% MD) in groups 1 and 2. B: Normal valve. C: Mitral valve with MVD. The valve with myxomatous degeneration shows diffuse and homogeneous thickening, distorted architecture, spongy layer expansion and proteoglycan accumulation, indicated by the bluish Movat staining (a: atrial layer, b: spongy layer, and c: fibrous layer). Bar = 500 µm. G1: group 1; G2: group 2; p: p value.

Fig. 3. Immunohistochemical analysis of inflammatory cells. A: Counts of $CD20^+$, $CD45^+$ and $CD68^+$ cells. The cells were identified with Aperio ImageScope View by their corresponding antigens (blue dots) in their respective histological images. B, D and F: normal mitral valve; C, E and G: mitral valve with myxoid degeneration. B and C: $CD20^+$ cells; D and E: $CD45^+$ cells. F and G: $CD68^+$ cells. Bar = 200 µm for B, D and F; 300 µm for C; and 400 µm for E and G. No.: number; mm^2: millimeters.

Fig. 4. Bacterial analysis by IHC, ISH and TEM. A: Counts of *Mycoplasma pneumoniae* (Mp), *Chlamydophila pneumoniae* (Cp) and *Borrelia burgdorferi* (Bb) antigens. B, D, F, H, J and L: Normal mitral valve. C, E, G, I, K and M: Mitral valve with myxomatous degeneration; B and C: IHC for Mp; D and E: Mp antigen identification (yellow dots) by Aperio analysis; F and G: IHC for Cp; H and I: Cp antigen identification (yellow dots) by Aperio analysis; J and K: IHC for Bb; L and M: Bp antigen identification (yellow dots) by Aperio analysis. N, O, P and Q: Transmission electron microscopy of bacterial elements (arrows). N: A microorganism with *Mycoplasma* characteristics was identified in the control group, with a long, single envelope membrane and a structure similar to a spirochete in the cytoplasm amid fragmented collagen tissue. O: This structure is consistent with *Borrelia*, long and winding, and was identified in the control group. P: Structures compatible with *Mycoplasma* were also identified in the MD group. Q: A microorganism with a wavy structure compatible with spirochete-containing organelles or endosymbionts (consistent with *Borrelia*) was observed in the MD group.

IHC: immunohistochemistry; ISH: *in situ* hybridization; TEM: transmission electron microscopy; Mp: *Mycoplasma pneumoniae*; Bb: *Borrelia burgdorferi*; G1: group 1; G2: group 2; µm^2: square micrometers; Ag: antigen; mm: millimeters. Bar = 50 µm for B-M. Bar = 1 µm for N-Q.

Fig. 5. MMP9 analysis. A: Counts of MMP9 antigens. B and D: Normal mitral valve. C and E: Mitral valve with myxomatous degeneration; B and C: IHC for MMP9; D and E: MMP9 antigen identification (yellow and orange dots) by Aperio analysis. IHC: immunohistochemistry; G1: group 1; G2: group 2; µm^2: square micrometers; Ag: antigen; mm: millimeters. Bar = 50 µm.

 Click here to download Figure Fig.1.tiff

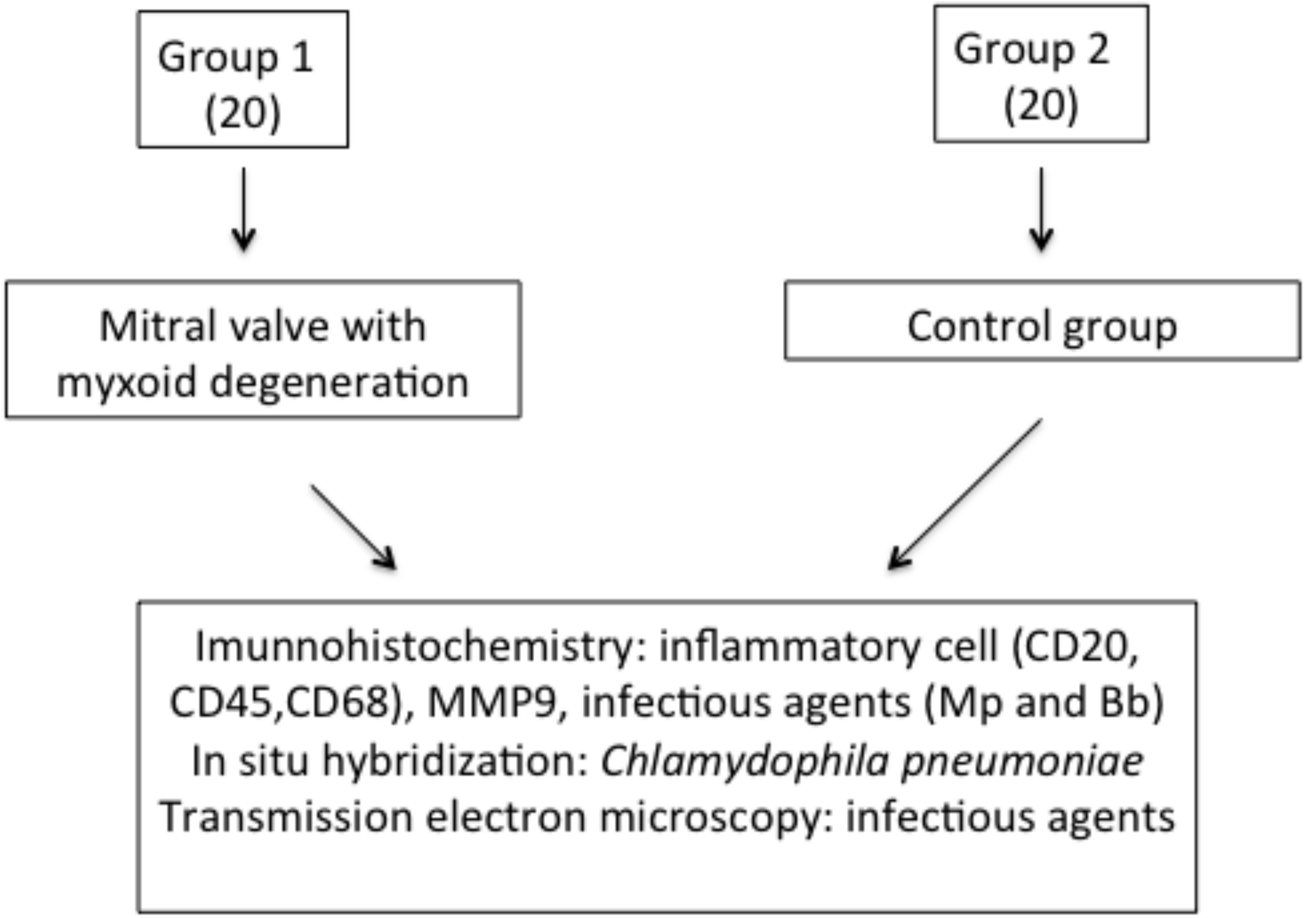

Figure 2

Click here to download Figure Fig.2.tif

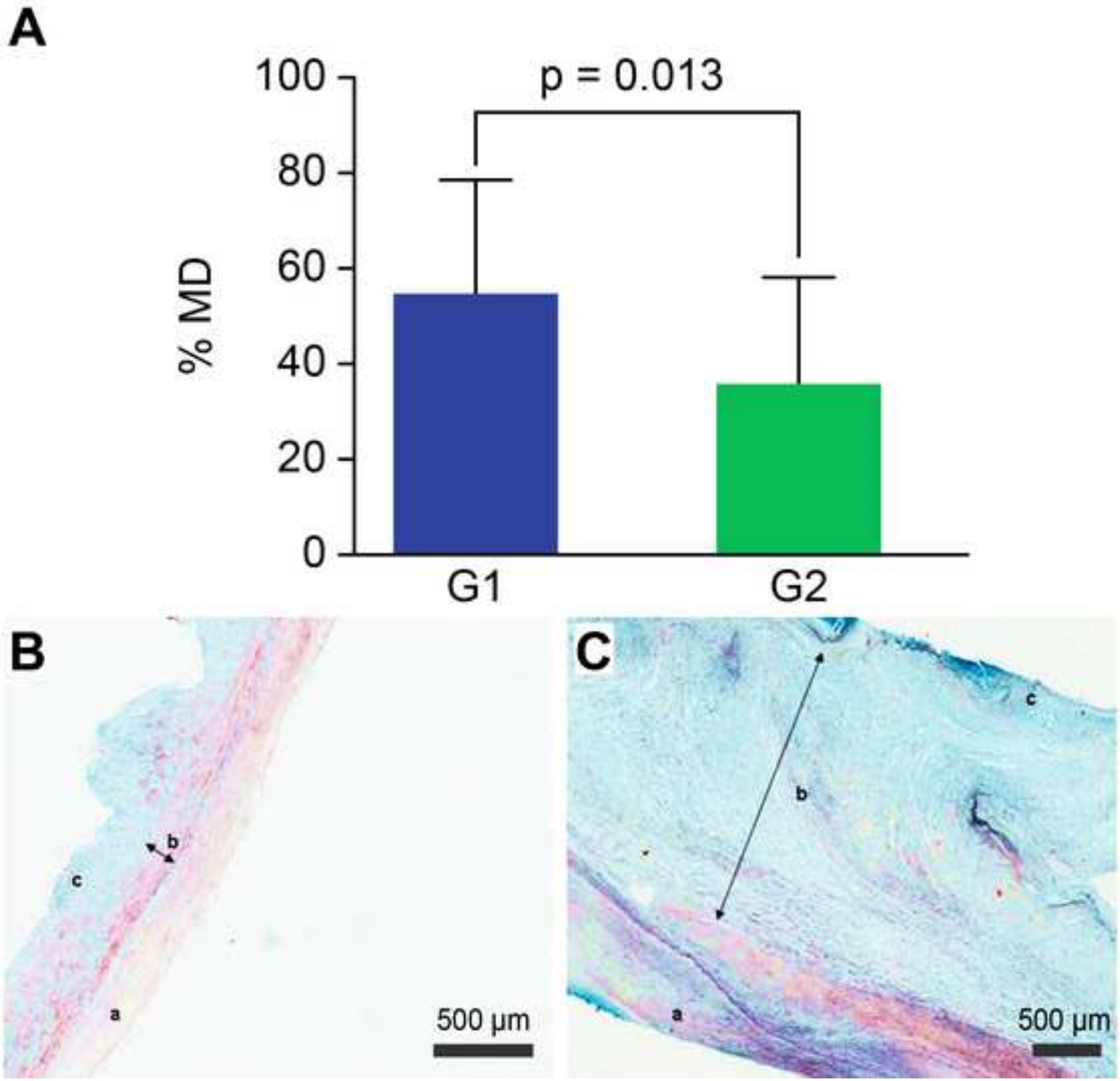

Figure 3

Click here to download Figure Fig.3.tif

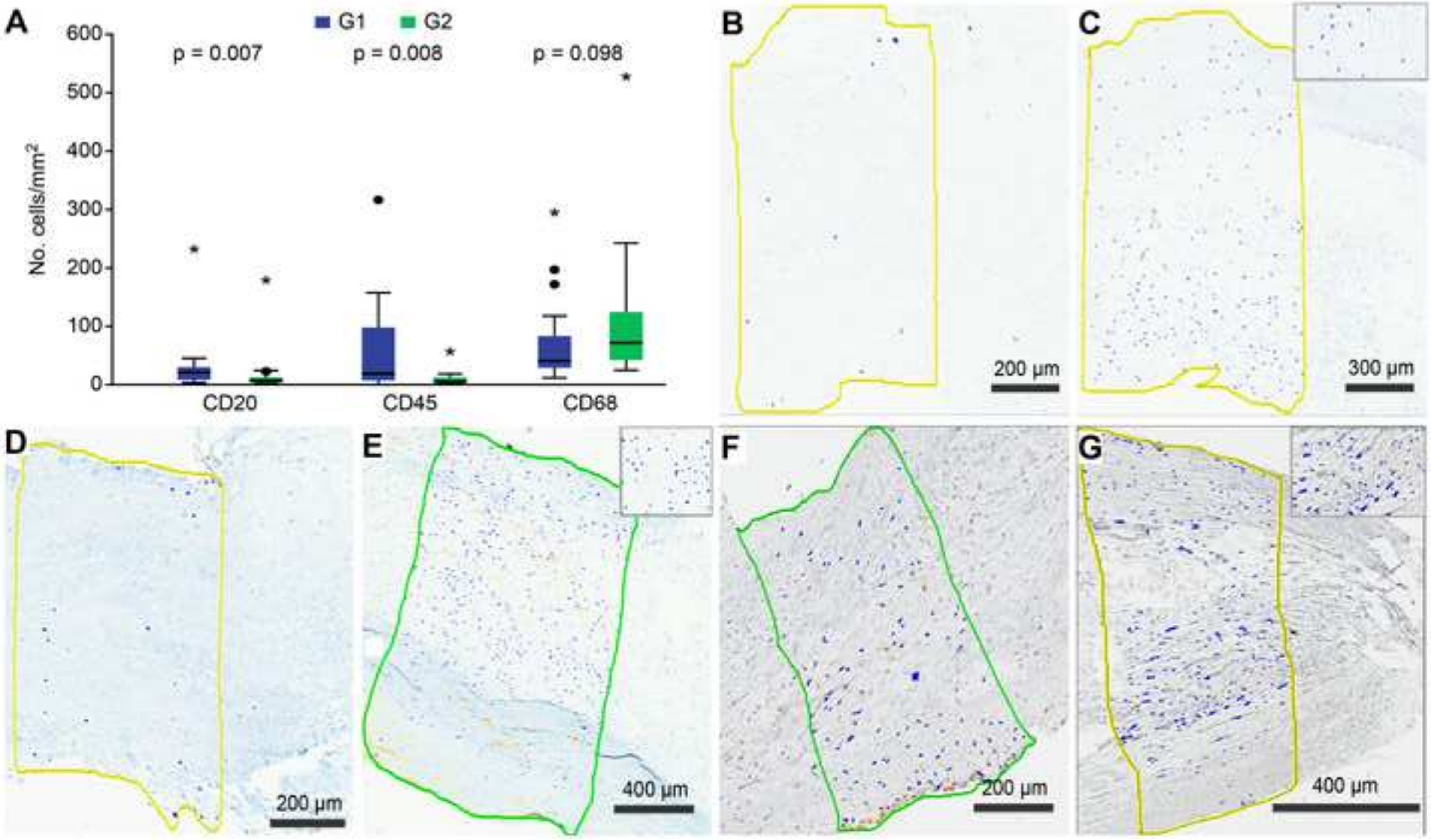

Figure 4

Click here to download Figure Fig.4.PDF

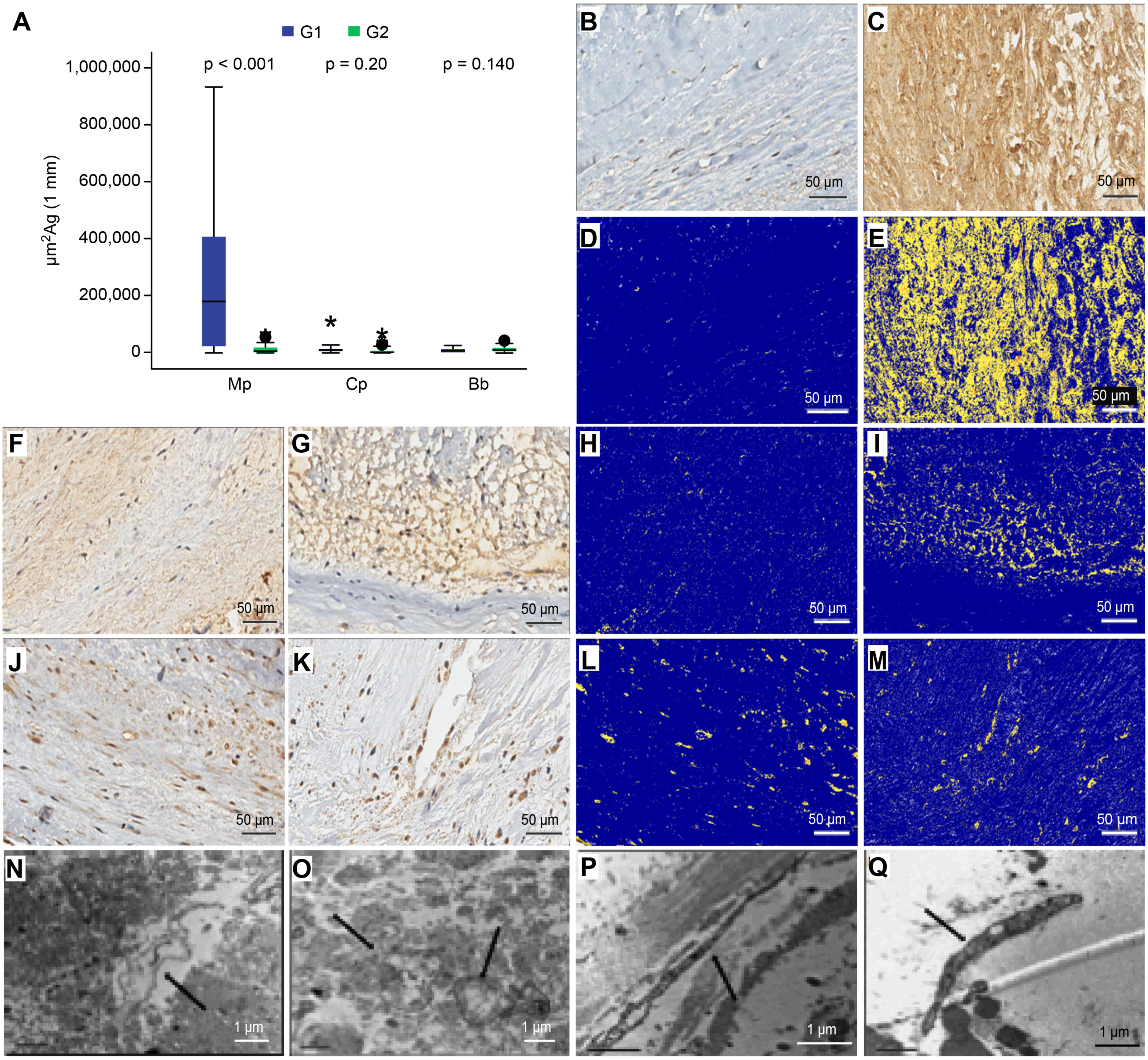

Figure 5 Click here to download Figure Fig.5.tif

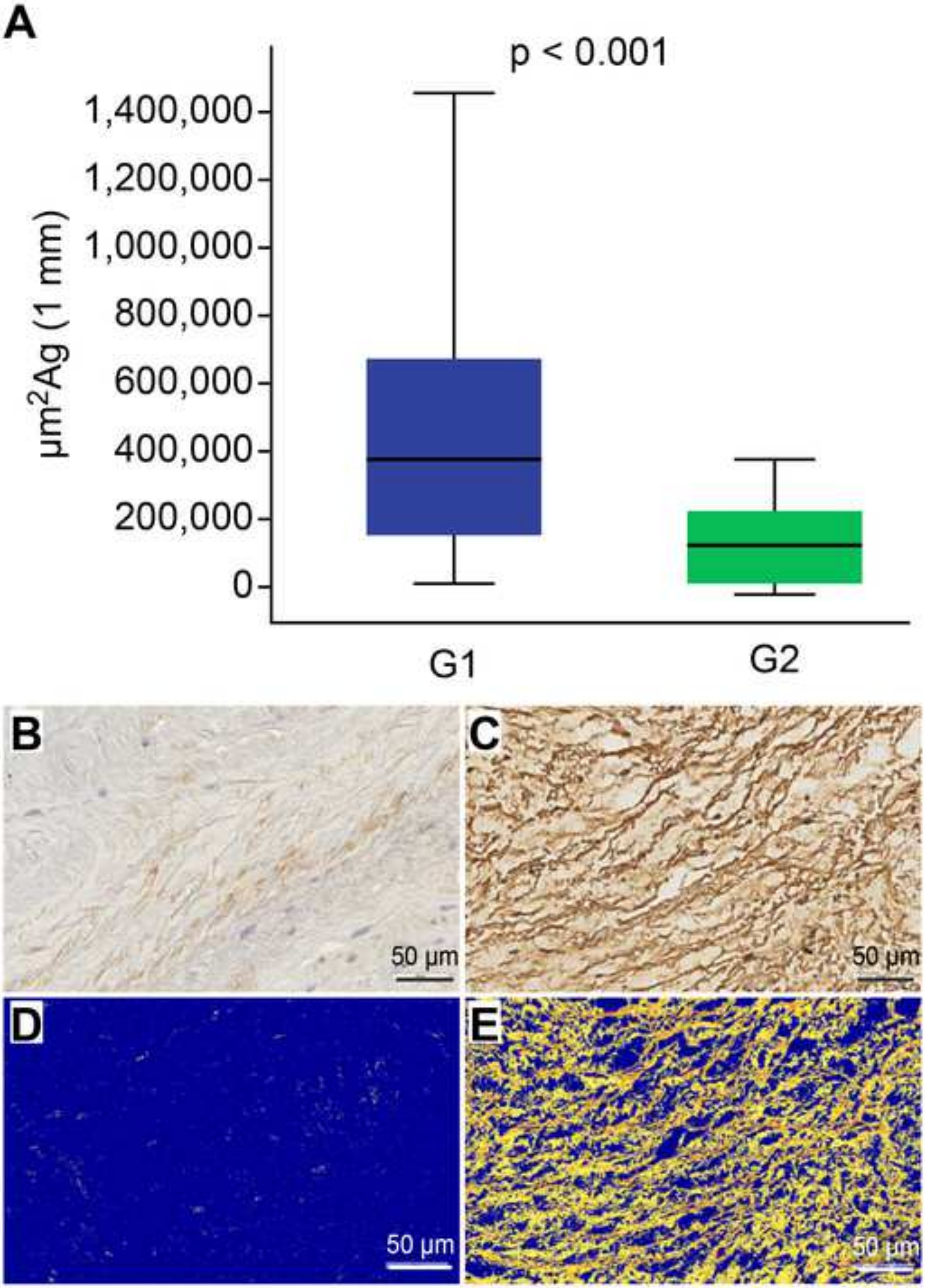

Supplementary Material

Click here to access/download
Supplementary Material
Supplementary Material.docx
