# Supplementary material for: Infectious agents is a risk factor for myxomatous mitral valve degeneration: A case control study
Source: BMC Infect Dis. 2017 Apr 21;17:297. doi: 10.1186/s12879-017-2387-8 (PMC5399830; doi:10.1186/s12879-017-2387-8)
Supplement: Additional file 1: — Quantification of antigens of Borrelia burgdorferi, Mycoplasma pneumoniae and MMP 9 containing only the cases with cardiovascular disease associated. (DOCX 15 kb) [file 12879_2017_2387_MOESM1_ESM.docx › Supplementary MaterialR4.docx]

**Supplementary Material**

Quantification of antigens of *Borrelia burgdorferi*, *Mycoplasma pneumoniae* and MMP 9 containing only the cases with cardiovascular disease associated.

| Bacteria  (µm^2^Ag/1 mm) | MD | CO | p |
| --- | --- | --- | --- |
| Bb | 3,821 (1,359-25,927) | 9,367 (2,991-14,703) | 0,32 |
| Mp | 213,513 (823-532,536) | 12.630 (364-66,129) | 0.013 |
| MMP9 | 349,678 (32,467-785,820) | 29,657 (3,012-379,092) | 0,037 |

Data are presented as the median value (25th, 75th percentile).

MD: myxomatous degeneration; CO: control; Mp: *Mycoplasma pneumoniae*; Bb: *Borrelia burgdorferi*; MMP9: metalloproteinase.
