# Supplementary figures and images for: Infectious agents is a risk factor for myxomatous mitral valve degeneration: A case control study
Source: BMC Infect Dis. 2017 Apr 21;17:297. doi: 10.1186/s12879-017-2387-8 (PMC5399830; doi:10.1186/s12879-017-2387-8)

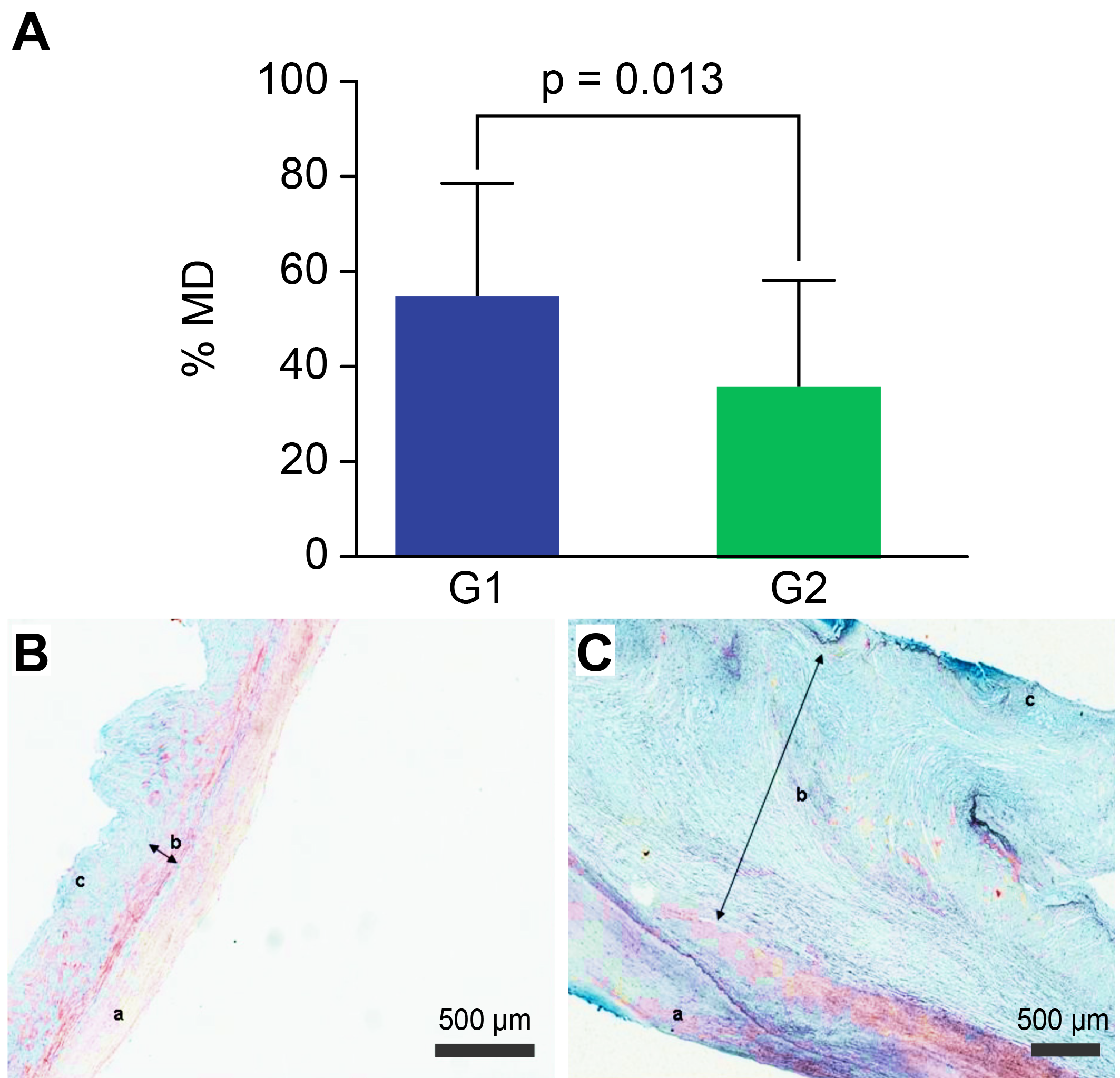

Supplement: Additional file 1: — Quantification of antigens of Borrelia burgdorferi, Mycoplasma pneumoniae and MMP 9 containing only the cases with cardiovascular disease associated. (DOCX 15 kb) [file 12879_2017_2387_MOESM1_ESM.docx › Fig.2R4.tif]

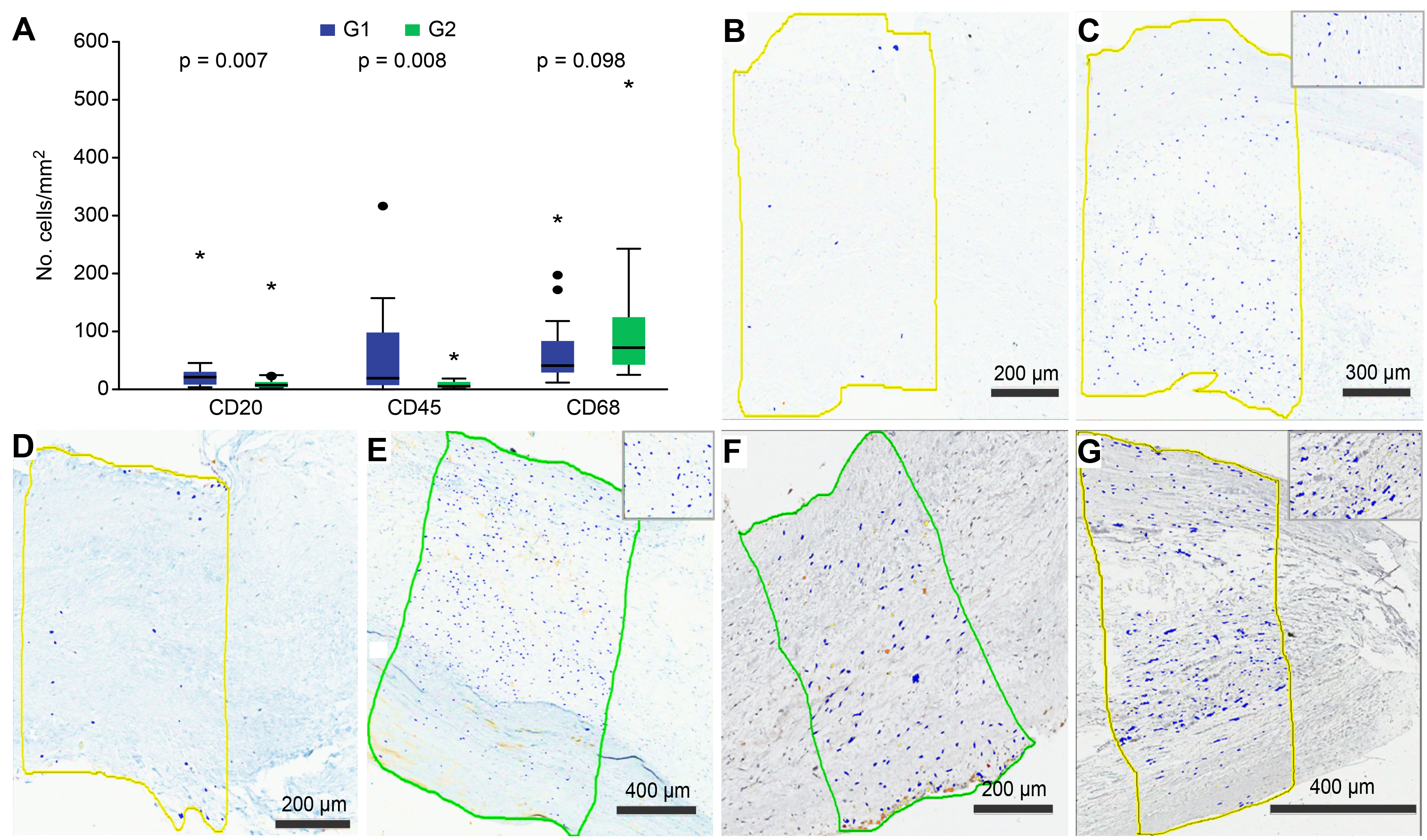

Supplement: Additional file 1: — Quantification of antigens of Borrelia burgdorferi, Mycoplasma pneumoniae and MMP 9 containing only the cases with cardiovascular disease associated. (DOCX 15 kb) [file 12879_2017_2387_MOESM1_ESM.docx › Fig.3R4.tif]

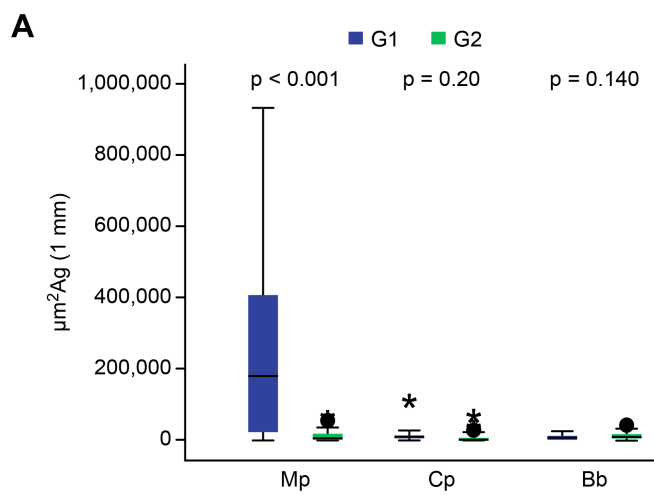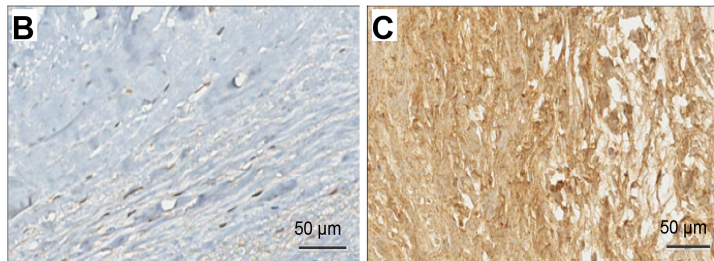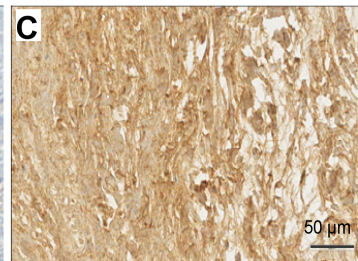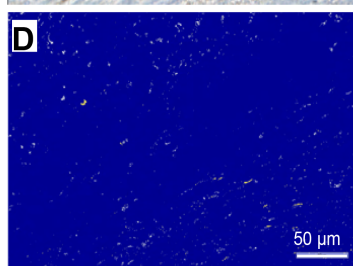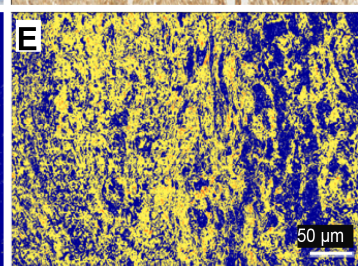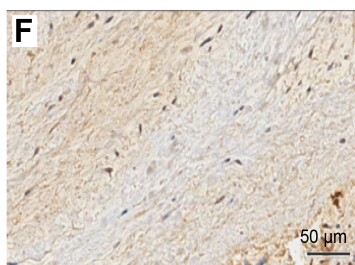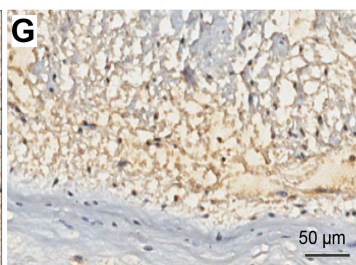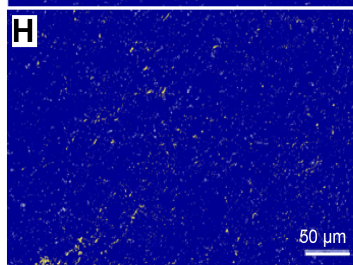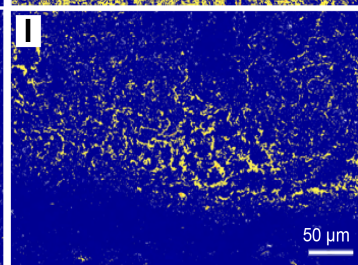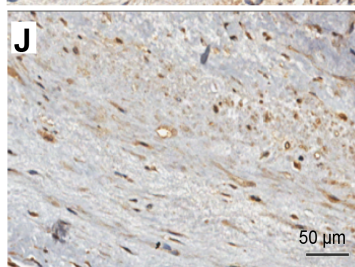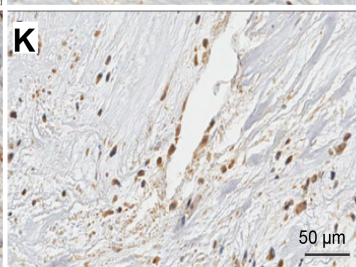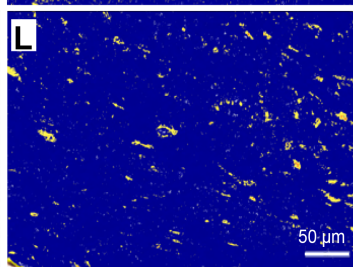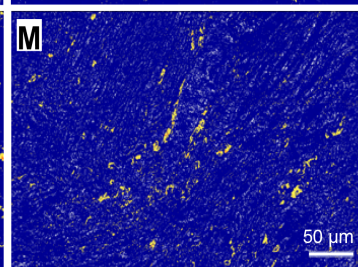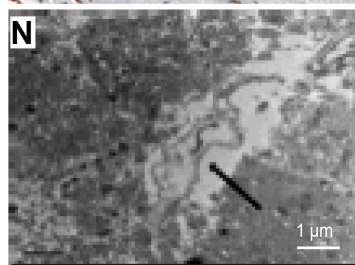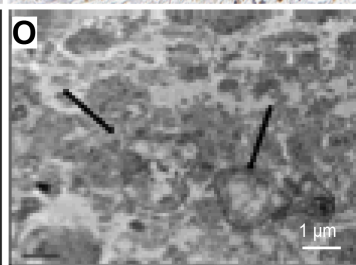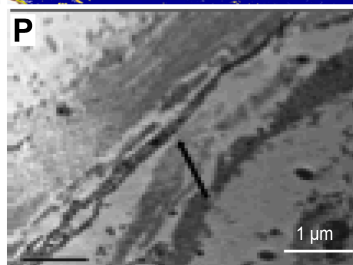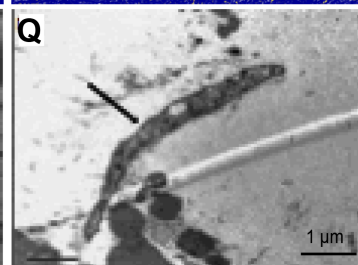

Supplement: Additional file 1: — Quantification of antigens of Borrelia burgdorferi, Mycoplasma pneumoniae and MMP 9 containing only the cases with cardiovascular disease associated. (DOCX 15 kb) [file 12879_2017_2387_MOESM1_ESM.docx › Fig.4R4.PDF]

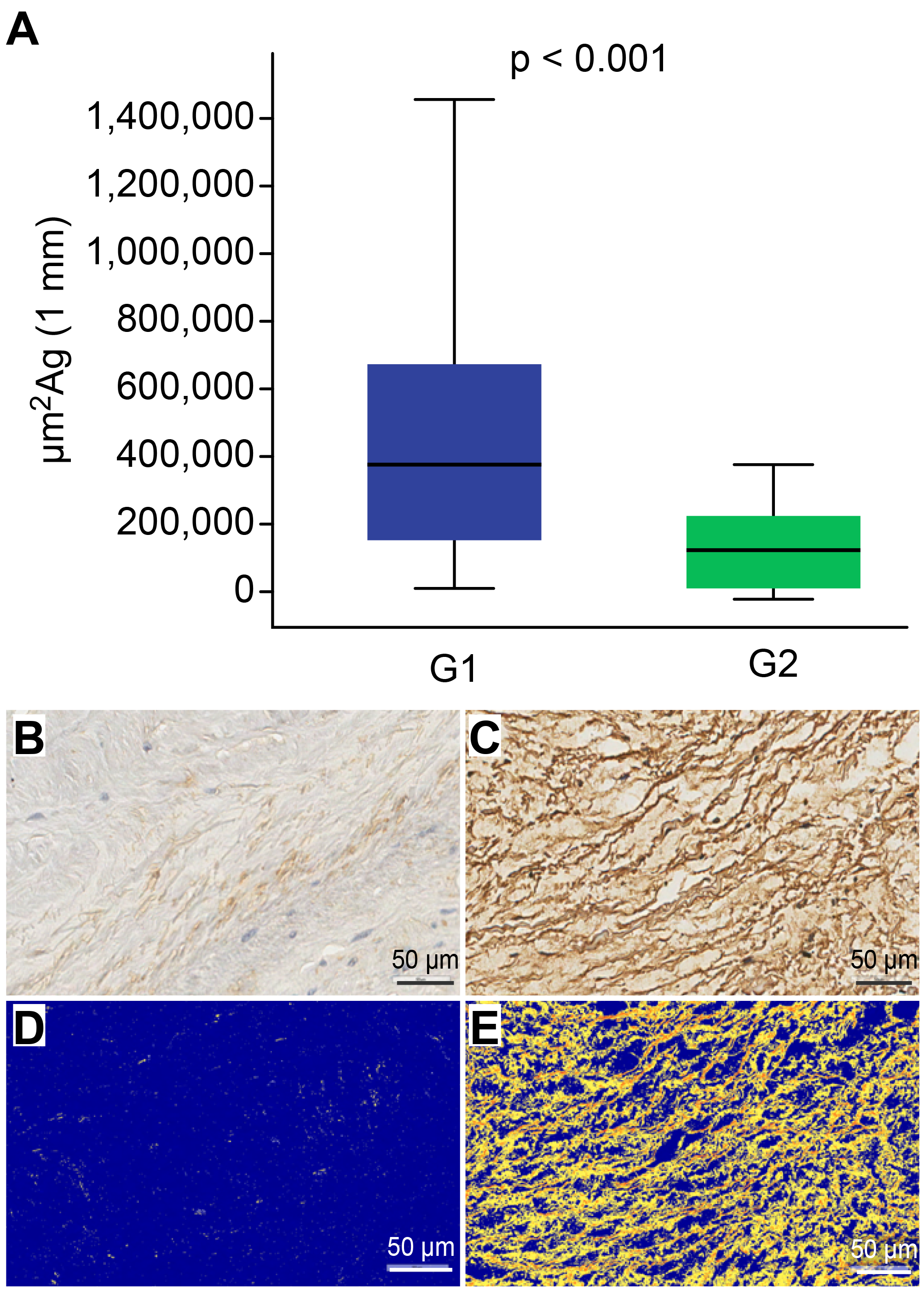

Supplement: Additional file 1: — Quantification of antigens of Borrelia burgdorferi, Mycoplasma pneumoniae and MMP 9 containing only the cases with cardiovascular disease associated. (DOCX 15 kb) [file 12879_2017_2387_MOESM1_ESM.docx › Fig.5R4.tif]
